# Correspondence

**Published:** 1910-02

**Authors:** 


### Correspondence.

HARTFORD, CONN., December 19, 1909.

*Dr. F. E. Daniel.*

MY DEAR DOCTOR: Will you be kind enough to review the statement, Document No. 48 of the Sixty-first Congress, under separate covers, in your excellent journal. This is the first transaction of a medical society ever published by the government, and is entitled to great prominence for this reason.

State in your *review that copies can be had by request of the United States Senators.* Already a very large edition is published, and another is called for.

Wishing you the best compliments of the season, believe me,

Very sincerely yours,

T. D. Crothers.

[See editorial.]

---

Burlington, N. J., January 17, 1910.

*Dr. F. E. Daniel.*

My Dear Doctor: I received the December and January numbers of your journal. The article on "Chronicles of the Octopus" is great; it is the finest thing I ever read. Every move made by the "powers that be" of the A. M. A. for the last few years has been a *bad* one. They are driving the old ship of the regular school onto the rocks. Here's to the "Red Back." Long may it wave, and be an exponent of the square deal.

Very kindly yours,

Eli G. Jones, M. D.,

Dartmouth, 1871.

["Chronicles of the Octopus" and "Song of the First Lord," in pamphlet form, for sale at this office $1.00; per dozen, or 10 cents each. Stamps.—Dr. Daniel.]

